# Asymmetricity Between Sister Cells of Pluripotent Stem Cells at the Onset of Differentiation

**DOI:** 10.1089/scd.2017.0113

**Published:** 2018-03-01

**Authors:** Shogo Nakamura, Atsushi Maruyama, Yuki Kondo, Ayumu Kano, Olga M. De Sousa, Masahiro Iwahashi, Bayar Hexig, Toshihiro Akaike, Jingyue Li, Yohei Hayashi, Kiyoshi Ohnuma

**Affiliations:** ^1^ Department of Bioengineering, Nagaoka University of Technology, Nagaoka, Japan.; ^2^ Department of Electrical, Electronics and Information Engineering, Nagaoka University of Technology, Nagaoka, Japan.; ^3^ Tokyo Institute of Technology, Yokohama, Japan.; ^4^ Faculty of Medicine, University of Tsukuba, Tsukuba, Japan.; ^5^ Department of Science of Technology Innovation, Nagaoka University of Technology, Nagaoka, Japan.

**Keywords:** asymmetrical division, single-cell-based analysis, pluripotent stem cells

## Abstract

Various somatic stem cells divide asymmetrically; however, it is not known whether embryonic stem cells (ESCs) divide symmetrically or asymmetrically, not only while maintaining an undifferentiated state but also at the onset of differentiation. In this study, we observed single ESCs using time-lapse imaging and compared sister cell pairs derived from the same mother cell in either the maintenance or differentiation medium. Mouse ESCs were cultured on E-cadherin-coated glass-based dishes, which allowed us to trace single cells. The undifferentiated cell state was detected by green fluorescent protein (GFP) expression driven by the *Nanog* promoter, which is active only in undifferentiated cells. Cell population analysis using flow cytometry showed that the peak width indicating distribution of GFP expression broadened when cells were transferred to the differentiation medium compared to when they were in the maintenance medium. This finding suggested that the population of ESCs became more heterogeneous at the onset of differentiation. Using single-cell analysis by time-lapse imaging, we found that although the total survival ratio decreased by changing to differentiation medium, the one-live-one-dead ratio of sister cell pairs was smaller compared with randomly chosen non-sister cell pairs, defined as an unsynchronized cell pair control, in both media. This result suggested that sister cell pairs were more positively synchronized with each other compared to non-sister cell pairs. The differences in interdivision time (the time interval between mother cell division and the subsequent cell division) between sister cells was smaller than that between non-sister cell pairs in both media, suggesting that sister cells divided synchronously. Although the difference in Nanog-GFP intensity between sister cells was smaller than that between non-sister cells in the maintenance medium, it was the same in differentiation medium, suggesting asymmetrical Nanog-GFP intensity. These data suggested that ESCs may divide asymmetrically at the onset of differentiation resulting in heterogeneity.

## Introduction

**FIG. 1.**
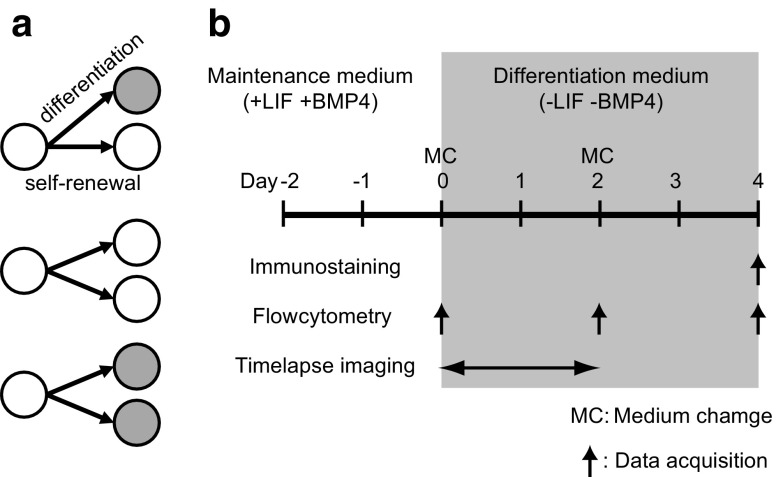
Schematics of experiments. **(a)** Schematic showing asymmetric and symmetric division. Asymmetric division (*upper*), and symmetric division (*middle* and *lower*) to produce an identical cell (*open circle*) or a differentiated cell (*closed circle*). **(b)** Experimental schematic of the experimental protocol.

Stem cells are defined by their ability to differentiate into specialized cells and to renew themselves (self-renewal). One strategy by which stem cells can accomplish these two tasks is asymmetric cell division, whereby each mother cell divides to generate one daughter cell with stem cell properties and another daughter cell that is capable of differentiation [1] (Fig. 1a).

It is proposed that pluripotent stem cells, including embryonic stem cells (ESCs) and induced pluripotent stem cells (iPSCs), both of which can differentiate into almost all cell types (pluripotency) and are maintained by infinite self-renewal in vitro, divide symmetrically to produce a homogeneous population [2]. However, it is not known whether ESCs can also divide asymmetrically. In support of this, it has been observed that ESCs easily and spontaneously differentiate. Thus, selection of undifferentiated cell colonies by picking is routinely performed in laboratories to maintain undifferentiated cell populations [3–6]. These facts suggest that ESCs may divide asymmetrically and undifferentiated cells can be selected by choosing a specific medium and/or by researchers.

In this study, using time-lapse imaging, we compared sister cells derived from single mother ESCs. We used the Nanog-reporter mouse ESCs expressing a green fluorescent protein (GFP) driven by the *Nanog* promoter (Nanog-GFP) to determine the differentiation state of the daughter cells [7]. *Nanog* is a self-renewal marker and is not expressed in differentiated cells. To observe single cells, we used culture dishes coated with E-cadherin to prevent the cultured cells from forming three-dimensional aggregates [8]. Single-cell culture on E-cadherin also reduces cell–cell interactions, which occur randomly and strongly affect cell differentiation [9]. To precisely control cell differentiation, we used a serum-free medium supplemented with leukemia inhibitory factor (LIF) and bone morphogenetic protein 4 (BMP4) to maintain the cells in an undifferentiated state (maintenance media). Non-directional differentiation was initiated by culturing cells in a serum-free medium lacking supplements (differentiation medium) [10,11].

Using this system, we compared the viability (live/dead), interdivision time, and differentiation state (fluorescent intensity of Nanog-GFP) of sister cells (Fig. 1b).

## Materials and Methods

### Culture of mouse ESCs

Mouse Nanog-GFP ESCs (RF8-NanogGIP No. 1A2) were provided by Dr. Yamanaka (Department of Stem Cell Biology, Institute for Frontier Medical Sciences, Kyoto University). The ESCs had stably incorporated the modified bacterial artificial chromosome (BAC, 200 kb) containing the mouse *Nanog* gene. A GFP-internal ribosome entry site (IRES)-puromycin resistance gene cassette was inserted into the 5′ untranslated region (UTR) of Nanog [7]. The maintenance medium consisted of ESF-basal medium (Cell Science & Technology Institute, Miyaghi, Japan) supplemented with 10 μg/mL insulin (Sigma-Aldrich, St. Louis, MO), 5 μg/mL transferrin (Sigma-Aldrich), 5 μL oleic acid-bovine serum albumin (BSA) solution (Sigma-Aldrich), 0.1 nM sodium selenite (Sigma-Aldrich), 100 nM 2-mercaptoethanol (Sigma-Aldrich), 100 nM ethanolamine (Sigma-Aldrich), 2% (v/v) B27 (GIBCO, Life Technologies), 1,000 U/mL LIF (ESGRO; Merck Millipore, Billerica, MA), and 2 ng/mL BMP4 (R&D Systems) [10,11]. To initiate differentiation, we used the same media without LIF and BMP4 (differentiation medium).

The ESCs were subcultured every 3–4 days in the maintenance medium. To select undifferentiated Nanog-GFP expressing ESCs, 0.75 μg/mL of puromycin was added to the culture dishes 1 day before subculturing [7]. All cells were removed from culture dishes using 0.02% (w/v) EDTA-4Na in phosphate-buffered saline (PBS), and 20,000 dissociated ESCs were plated onto 35-mm-diameter tissue culture dishes coated with 20 μg/cm^2^ collagen Type I-A (Nitta Gelatin, Osaka, Japan). All culture systems were incubated in 5% CO_2_ at 37°C, and the medium was replaced every second day.

### Immunostaining and flow cytometric analysis

For immunostaining, the ESCs were fixed in 10% formaldehyde, permeabilized with 0.1% Triton X-100, blocked with 1% BSA, and then stained with an anti-Oct3/4 antibody (rabbit polyclonal IgG 1:100; Santa Cruz Biotechnology, Dallas, TX) or an anti-FGF5 antibody (rabbit polyclonal IgG 1:100; Santa Cruz Biotechnology) as primary antibodies. Primary antibody binding was visualized using AlexaFluor 546-conjugated anti-rabbit polyclonal IgG (1:2,000; Invitrogen, Carlsbad, CA). Nuclei were stained with 0.4 μM 4′,6-diamidino-2-phenylindole (DAPI; Wako Pure Chemical Industries, Osaka, Japan). Micrographs were obtained using a BZ-8100 microscope (Keyence, Osaka, Japan).

For flow cytometry analysis, all cells were removed from culture dishes using 0.02% (w/v) EDTA-4Na in PBS, and then 0.1 μg/mL propidium iodide (PI; Wako Pure Chemical Industries) was added to aid identification of dead cells. A JSAN flow cytometer (Bay Bioscience Co., Kobe, Japan) was used for data acquisition.

### Time-lapse microscopy

One-thousand dissociated ESCs per well were plated into four-well glass-based dishes coated with a fusion protein of E-cadherin and the Fc domain of IgG [8]. To make the four-well glass-based dish, a heat-cured silicone elastomer, polydimethylsiloxane (PDMS; Sylgard 184 Silicone Elastomer Kit; Dow Corning Toray Co., Tokyo, Japan) was punched to create four 8-mm-diameter holes and then attached to a glass cover slip (Matsunami Glass Ind., Osaka, Japan) with non-cured PDMS. The ESCs were then cultured in the maintenance medium for 2 days (days 2–0). On day 0, the medium was replaced with either fresh maintenance or differentiation medium (Fig. 1). The dish was then placed in the culture chamber. The culture chamber was placed on an inverted microscope (ECLIPSE Ti-E; Nikon Instech Co., Tokyo, Japan) equipped with a motor-driven X/Y stage (BIOS-105T; Sigmakoki Co. Ltd., Tokyo, Japan), a thermal insulation system (Nikon Instech Co.), and a cooled CCD camera (ORCA-ER; Hamamatsu Photonics, Shizuoka, Japan). The culture chamber was supplied with humidified 5% CO_2_ and 95% air and maintained at 37°C. The microscope system was controlled using μManager software (https://micro-manager.org). To construct lineage trees, including cell division timing and survival information, and measure fluorescent intensities of Nanog-GFP, the cells were manually tracked and outlined based on both GFP images and phase contrast images using Image J software (NIH, MD). Total brightness was measured as a product of the cell body area (pixel counts) and average brightness (8-bit gray scale). Statistical analyses were performed using R software (version 2.15.3). To compare two distributions, *P* values were calculated based on chi-square tests using R software without multiple test compensation. Because a distribution with many zero values causes an inaccuracy warning in the R software, the range data denoted by the arrows were used (Fig. 5 and Supplementary Fig. S2).

## Results

### Cell population studies

First, we observed the state of cells during maintenance of the undifferentiated state and during the onset of differentiation using cell population studies. Dissociated mouse ESCs expressing Nanog-GFP were plated and cultured in the maintenance medium, following which the medium was replaced with either fresh maintenance or differentiation medium. Next, cells were observed to determine if they were in an undifferentiated or differentiated state (Fig. 1b).

**FIG. 2.**
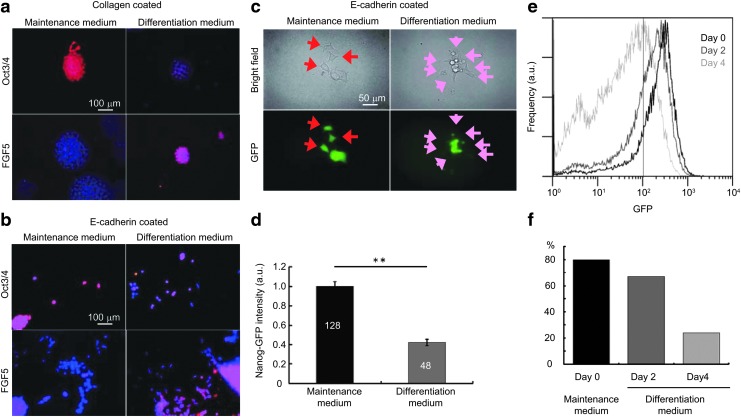
Differentiation of ESCs. **(a, b)** Immunostaining images of Oct3/4 and FGF5 for ESCs grown on collagen plates **(a)** or E-cadherin-coated plates **(b)** with two types of medium: maintenance medium and differentiation medium. The nuclei were stained with DAPI (*blue*). **(c)** Microscopic images of bright field and Nanog-GFP fluorescence for living ESCs on E-cadherin-coated plates in maintenance medium and differentiation medium. **(d)** Mean GFP intensity in individual ESCs from **(c)**. GFP was quantified by multiplying the cell body area (pixel counts) by the average brightness (256 grayscale) (**P < 0.01, *t*-test). The *numbers* in the columns represent the numbers of cells measured. **(e, f)** Flow cytometry profile **(e)** and percentage **(f)** of Nanog-GFP-positive ESCs on collagen-coated dishes on day 0 in the maintenance medium, day 2 in the differentiation medium, and day 4 in the differentiation medium. DAPI, 4′,6-diamidino-2-phenylindole; ESCs, embryonic stem cells; GFP, green fluorescent protein.

Immunocytochemistry of cells cultured on either collagen- or E-cadherin-coated dishes revealed that, although most cells expressed Oct3/4 (a marker of undifferentiated cells) in the maintenance medium, most cells expressed FGF5 (a marker of early differentiated cells) in the differentiation medium (Fig. 2a, b). However, some cells in the differentiation medium also expressed Oct3/4. Using live-cell fluorescent microscopy, we also detected more reduction in the fluorescent intensity of Nanog-GFP in the differentiation medium than in the maintenance medium. However, this loss of Nanog-GFP intensity in the differentiation medium was limited to a proportion of the cell population (Fig. 2c, d). The flow cytometry profile showed a narrow peak for cells in the maintenance medium, which became broader after changing to the differentiation medium (Fig. 2e, f). The width change in flow cytometry profile was also confirmed using Nanog-GFP mouse iPSCs and a different medium (Supplementary Fig. S1; Supplementary Data are available online at www.liebertpub.com/scd). Taken together, these data demonstrated that ESCs were more heterogeneous at the onset (first few days) of differentiation than when they were maintained in the undifferentiated state.

### Single-cell observations

Next, we confirmed whether ESCs could be cultured as single cells on E-cadherin-coated dishes. Fixed cell images showed that most cells formed large colonies in both the maintenance and differentiation media on collagen-coated dishes (Fig. 2a). However, on E-cadherin-coated dishes, although some cells formed large colonies, others adhered to the dish as single cells in both culture media (Fig. 2b). A similar trend was also observed using live-cell imaging (Fig. 2c). These results suggested that single cells could be observed in our system.

**FIG. 3.**
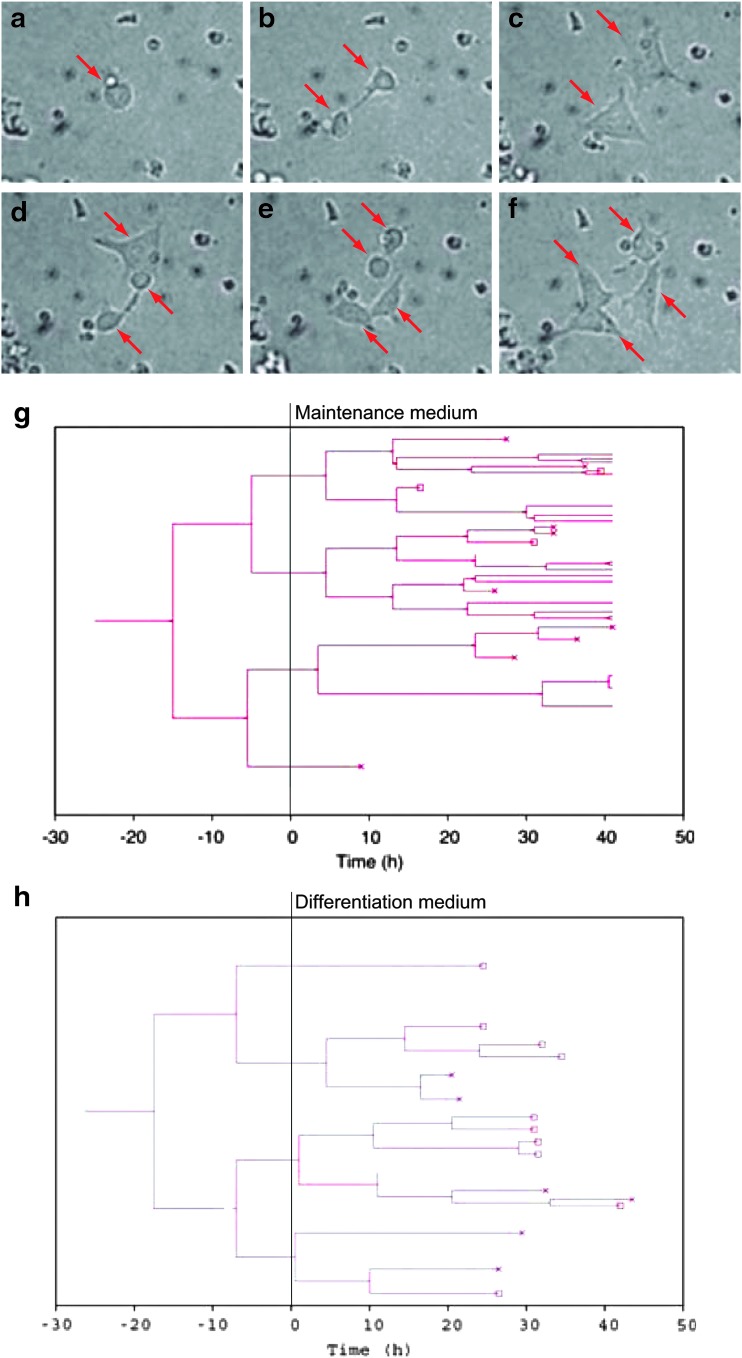
Time-lapse imaging and tree diagram. **(a–f)** Time-lapse video microscopy of ESCs on E-cadherin-coated wells in the maintenance medium. *Red arrows* indicate the cells. **(g, h)** Tree diagrams of ESCs on E-cadherin-coated well in maintenance medium **(g)** and differentiation medium **(h)**. The *red crosses* indicate cell death.

Using our single-cell observation system, time-lapse images were obtained at two frames per hour for 2 days (Figs. 1b and 3). Although a proportion of the dividing cells remained attached after division to form aggregates, others separated fully and remained as single cells, which were easily distinguishable from each other (Fig. 3a and Supplementary Movies S1 and S2). These single cells were manually tracked to record their history of divisions, survival, and fluorescent intensities. From the traced data, lineage trees were constructed (Fig. 3g, h). A maximum of six sequential divisions were observed for cells in both media (Fig. 3g, h).

Synchronous divisions were observed for cells in the maintenance medium (Fig. 3g). For example, the mother cells divided to produce two daughter (sister) cells at −16 h. These sister cells divided synchronously at −6 h to produce four granddaughter cells, and three of the four granddaughter cells divided synchronously around +3 h to produce great-granddaughter cells, although one of these was not viable (Fig. 3g). Synchronous divisions between sister cells were also observed in the differentiation medium (after time 0; Fig. 3h). These synchronicities became weaker as sequential division proceeded. These observations suggested that the similarity in interdivision time between the sister cells was higher than that between distantly related cells.

### Difference in viability between sister cells

**FIG. 4.**
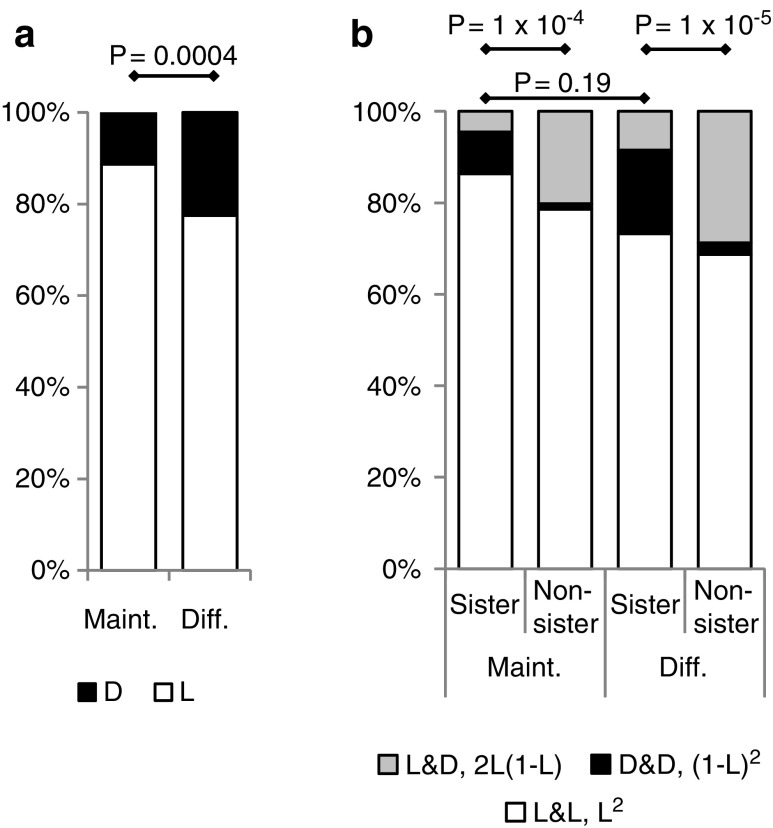
Sister cell analysis of cell viability. Living and dead cells were measured in maintenance medium (88 cells) and differentiation medium (71 cells). **(a)** Total viability was calculated from all the 176 and 142 daughter cells. L: ratio of live cells that produced granddaughter cells. D: ratio of cells that were dead before producing granddaughter cells (1 **−** L). **(b)** Observed viability of sister cell pairs, and calculated viability from total cell viability by assuming randomly chosen non-sister pairs. L&L, D&D, and L&D indicate both cells lived, both cells died, and one cell lived, but the other died in sister cell pairs, respectively. 2L(1 **−** L), (1 **−** L)^2^, and L^2^ were calculated using L by assuming a randomly chosen non-sister pair. *P* values were calculated based on a chi-square test of the ratio of D and L. **(a)** The ratio of L&D compared to the others **(b)**, and the ratio of 2L(1 **−** L) compared to the others. **(b)** *P* values were calculated based on a chi-square test without compensation for the multiple tests.

First, we focused on cell viability to understand the similarity between sister cells derived from the same mother cells. We obtained data from 88 or 71 sister cell pairs after changing to fresh maintenance medium or differentiated medium, respectively. The total survival ratio (L) was calculated from the total number of cells in each medium following one division (ie, 176 and 142 cells in the maintenance and differentiation medium, respectively). L for the differentiated cells (77%) was lower than that for undifferentiated cells (89%) (Fig. 4a). Next, we compared the survival ratios of sister cell pairs when both cells lived (L&L), both cells died (D&D), and one cell lived and the other one died (L&D). The L&D category represented asynchronicity between sister cells. Although the L&D ratio of sister pairs in the maintenance medium (4.5%) was smaller than that in the differentiated medium (8.5%), no significant difference was observed between the media types (Fig. 4b). These results suggested that although the medium change to differentiation medium was harmful for cells, it affected both sister cells equally.

To quantify synchronicity in survival between sister cells, we compared the L&D ratios of sister cells to those of randomly chosen non-sister cell pairs, which were calculated using L. Randomly chosen non-sister cell pairs were used as an unsynchronized control. For example, if (A1, A2) and (B1, B2) were two sister cell pairs, the non-sister cell pairs were (A1, B1), (A1, B2), (A2, B1), and (A2, B2). Since the randomly chosen non-sister cells may potentially survive independent of each other, the probability of both living, both dying, and one living and one dying were L^2^, (1 − L)^2^, and 2L(1 − L), respectively. If the L&D ratio of a sister pair and the 2L(1 − L) probability of non-sister cells were the same, the sister cells survived randomly without any synchronization. If L&D of a sister pair was smaller or larger than the 2L(1 − L) probability of non-sister cells, the survival of sister cells was positively or negatively synchronized, respectively. We found that the L&D ratio of sister pairs was significantly smaller than the 2L(1 − L) probability of non-sister cells in both media, indicating that they were positively synchronized (Fig. 4b). These results suggested that the sister cells shared similar properties even after dividing from the mother cell.

### Differences in interdivision time and Nanog-GFP fluorescent intensity between sister cells

**FIG. 5.**
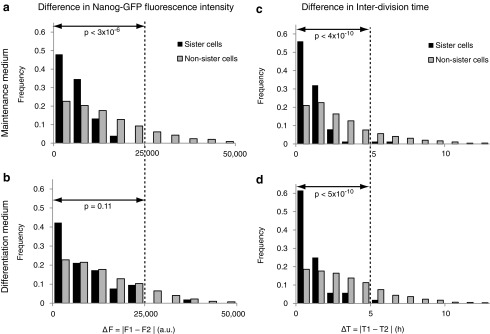
Sister cell analysis of interdivision time and Nanog-GFP intensity. Sister cell pairs, 76 cells and 52 cells, were measured in maintenance medium **(a, c)** and differentiation **(b, d)** medium, respectively. **(a–d)** Distribution of differences in fluorescence intensities between two cells **(a, b)** and the interdivision time of two cells **(c, d)** in maintenance medium **(a, c)** and differentiation medium **(b, d)**. *P* values were calculated based on a chi-square test using the range of data, denoted by the *arrow*, with the other data not being used to avoid inaccuracy.

To quantify the similarity between two cells, the absolute differences between Nanog-GFP intensity (ΔF = |F_1_ − F_2_|) and the interdivision time (ΔT = |T_1_ − T_2_|) were calculated, where F_1_ and F_2_ were the fluorescent intensities and T_1_ and T_2_ were the interdivision times of two cells, respectively. The interdivision time of a cell is the time interval between the start point of the cell following mother cell division and the end point of the cell when it subsequently divides. Fluorescence intensity was measured one frame before the next division. The interdivision time is the time period between sequential divisions. The distribution of these differences between sister cells was compared to that of randomly chosen non-sister cells (Fig. 5). ΔT and ΔF values that were smaller for the sister pairs than those for the randomly chosen non-sister pairs indicated a higher similarity between sister cells.

In the maintenance medium, higher peaks at zero and shorter right-tails were detected for the ΔF and ΔT of sister cells than for the randomly chosen non-sister cells (Fig. 5a, c). Significant differences were detected for both ΔF and ΔT (Fig. 5a, c; chi-square test, *P* < 3 × 10^−6^ and *P* < 4 × 10^−10^, respectively). These data suggested that in the maintenance medium, the sister cells were more similar to each other than to non-sister cells.

In the differentiation medium, higher peaks at zero and shorter right-tails were also detected for the ΔF and ΔT of sister cells than for the randomly chosen non-sister cells (Fig. 5b, d). However, although there was a significant difference in ΔT (Fig. 5d, chi-square test, *P* < 5 × 10^−10^), we failed to reject the null hypothesis that the ΔF distribution of a sister cell pair and that of a randomly chosen non-sister cell pair was the same, that is, we could not detect a significant difference in ΔF between sister cells and between randomly chosen non-sister cells (Fig. 5b; chi-square test, *P* = 0.11). Similar results were obtained for ΔF using the Nanog-GFP mouse iPSC line (Supplementary Fig. S2). These data show that the sister cells were more similar in ΔT, but not in ΔF than the non-sister cells. Taken together, these data suggested that when the medium was changed to differentiation medium, although the interdivision times of the sister cells were more similar to each other than to non-sister cells, Nanog-GFP intensity of the sister cells was different from each other and similar to that of non-sister cells.

## Discussion

In this study, Nanog-GFP mouse ESCs were cultured on E-cadherin-coated dishes in a serum-free culture medium to compare sister ESCs. Using this approach, we were able to trace single ESCs and analyze the differences between sister cells in the maintenance and differentiation media. Both immunostaining and live-cell analysis, including time-lapse imaging, showed that our culture system was suitable for single-cell tracking of mouse ESCs in maintenance and differentiation media. Our single-cell culture system could be easily controlled through changes in the culture medium compared to an ordinary aggregate culture [8]. In spherical cell aggregates, referred to as embryoid bodies, there are numerous cell–cell interactions that lead to heterogeneous differentiation, making the system difficult to control [12].

Although our system did not suffer from this disadvantage, it suffered from the limitation that we could only analyze a small number of cells using single-cell image analysis because of aggregate formation, cell division, and the presence of other cells. We plated one-thousand dissociated ESCs; however, we analyzed <70 cells in one experiment, indicating that a very low percentage (<7%) of cells was analyzed. The data obtained from these few cells might not reflect the behavior of the whole cell population. Large-scale data acquisition and automated analysis are required to overcome this limitation [13–15], which is our future goal.

**FIG. 6.**
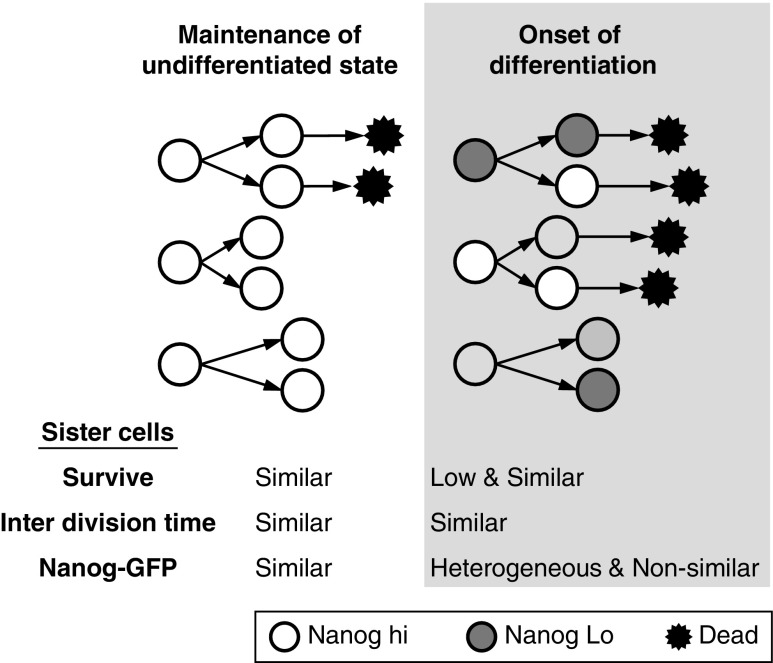
Schematic summarizing the cell division patterns.

Changing from that maintenance medium to differentiation medium changed the distribution of Nanog-GFP intensity between cells. It should be noted that Nanog-GFP intensity reflects not only *Nanog* expression but also the basal cell function, such as GFP degradation and the cell size, since the half-life of fluorescent proteins is more than 10 h in mouse ESCs [16,17], suggesting that Nanog-GFP intensity may reflect cell state associated with ESC maintenance. Flow cytometry analysis revealed that the peak width of Nanog-GFP distribution in the cells became more heterogeneous when the medium was changed to differentiation medium. Moreover, by single-cell analysis using time-lapse imaging, we found that sister cells became different from each other, as did non-sister cells, when the medium was changed to the differentiation medium (Fig. 6). Similar changes in Nanog-GFP induced by differentiation were also confirmed using mouse iPSCs. Thus, it is possible that an asymmetrical division occurs at the onset of differentiation to produce heterogeneous cell population.

There are two possible explanations for this asymmetry at the onset of differentiation. The first possibility is that ESCs start to stochastically differentiate into different linages, which may have different Nanog-GFP levels, after the induction of differentiation. ESCs derived from the inner cell mass in an early embryo. However, unlike ESCs, the cells of the inner cell mass do not self-renew perpetually, but do continually differentiate [2]. Since the differentiation medium does not contain LIF or BMP4, both of which force the ESCs toward a self-renewal state, the ESCs are able to adopt a transient state (like in the embryo), in which the cells divide both symmetrically and asymmetrically to produce multiple lineages [2,11]. Moreover, the primed state ESCs, which are postimplantation epiblast stem cells and considered to be in a transient state toward differentiation, express less Nanog than naive state ESCs [18–20]. A proportion of the cells can be in the primed state by differentiation. Since cells differentiate into multiple lineages in the embryo, there is a possibility that we detected the onset of multimodal asymmetry, although we cannot clearly detect discriminable subpopulations.

The second possibility is that temporal fluctuation in the cell state is increased by differentiation [21–23]. It has been reported that the expression of ESC-related genes, such as *Nanog*, *Hes1*, *Rex1*, and *Zscan4*, fluctuates over time to produce heterogeneity, and this fluctuation may be important in maintaining ESC pluripotency as well as in determining the direction of differentiation [16,24–27]. Fluctuations in *Nanog* levels are well known and can be important in maintaining ESC pluripotency, which is open to argument [17,22,23,27]. The phase of oscillation in *Hes1* expression may affect cell fate decision [21,26]. Zinc finger and SCAN domain containing 4 (ZSCAN4), which is specifically expressed in the two-cell stage mouse embryo and transiently and intermittently expressed in ESCs, are required for telomere elongation and genomic stability, and are thus considered to be important for maintaining ESCs [16,28–30]. Moreover, in general, the transient fluctuation is elicited as a response to a sudden change in the external environment (eg, the culture medium being changed to a differentiation medium). To test these two possibilities, faster and/or more sophisticated lineage tracking systems will be required (c.f., Bhadriraju et al. [13], Smith et al. [17], Frieda et al. [31]). However, in both cases, selection might occur to eliminate undifferentiated cells and to select differentiated cells during differentiation [5,32].

Understanding and controlling the state of ESCs are important because these cells, as well as iPSCs, are a promising resource for regenerative medicine and drug screening [33]. We believe that dynamic analyses, like those presented on this study, will be important for the future application of ESCs and iPSCs.

## Supplementary Material

Supplemental data
