# Supplementary material for: Asymmetricity Between Sister Cells of Pluripotent Stem Cells at the Onset of Differentiation
Source: Stem Cells Dev. 2018 Mar 1;27(5):347–54. doi: 10.1089/scd.2017.0113 (PMC5833898; doi:10.1089/scd.2017.0113)
Supplement: Supplemental data [file Supp_Data.zip › Supp_Data.pdf]

Supplementary Data

Supplementary Materials and Methods

Culture of mouse induced pluripotent stem cells

Mouse Nanog-green fluorescent protein (GFP)-induced pluripotent stem cells were generated using Sendai Virus Vectors and were confirmed to transmit to the germline [1,2]. The maintenance medium consisted of Stemsure Dulbecco's modified Eagle's medium (Wako Pure Chemical Industries, Osaka, Japan) supplemented with 1% (v/v) N2 supplement (Wako Pure Chemical Industries), 2% (v/v) B27 (GIBCO, Life Technologies), 1,000 U/mL leukemia inhibitory factor (LIF; Wako Pure Chemical Industries), and 1 μM PD0325801 (Wako Pure Chemical Industries), and 3 μM CHIR99021 (Wako Pure Chemical Industries) as LIF2i medium. To initiate differentiation, we used the same medium without LIF, PD0325801, or CHIR99021. The miPSCs were subcultured every 4–6 days in the maintenance medium. To select undifferentiated or Nanog-GFP expressing miPSCs, 1 μg/mL of puromycin (Wako Pure Chemical Industries) was added to the culture dishes 2 days after subculturing. All cells were removed from culture dishes using Accutase (Nacalai Tesque, Kyoto, Japan), and 25,000 dissociated miPSCs were plated onto 35-mm-diameter tissue culture dishes coated with gelatine (Wako Pure Chemical Industries). All culture systems were incubated in 5% CO_2 at 37°C, and the medium was replaced every second day.

Flow cytometry analysis

All cells were removed from culture dishes using Accutase, and then 0.1 μg/mL propidium iodide (PI; Wako Pure Chemical Industries) was added to allow for detection of dead cells. A FACSVerse flow cytometer (BD Biosciences, Franklin Lakes, NJ) was used for data acquisition.

Time-lapse microscopy

One-thousand dissociated ESCs per well were plated into four-well glass-based dishes (Nalge Nunc, Rochester, NY) coated with 3 $\mu g/cm^2$ of E-cadherin-IgG Fc domain fusion proteins (R&D Systems, Minneapolis, MN). The ESCs were cultured in maintenance medium for 2 days (days 2–0). On day 1, the culture chamber was placed on an inverted microscope (BX-X710; Keyence, Osaka, Japan) equipped with a thermal insulation system (Tokai Hit, Shizuoka, Japan). The culture chamber was supplied with humidified 5% CO_2 and 95% air and maintained at 37°C. On day 0, the medium was replaced with either fresh maintenance or differentiation medium. Time-lapse imaging was continued until day 4 (96 h).

Supplementary References

1. Nishimura K, T Kato, C Chen, L Oinam, E Shiomitsu, D Ayakawa, M Ohtaka, A Fukuda, M Nakanishi and K Hisatake. (2014). Manipulation of KLF4 expression generates iPSCs paused at successive stages of reprogramming. Stem Cell Reports 3:915–929.
2. Nishimura K, S Aizawa, FL Nugroho, E Shiomitsu, YT Tran, PL Bui, E Borisova, Y Sakuragi, H Takada, et al. A role for KLF4 in promoting the metabolic shift via TCL1 during induced pluripotent stem cell generation. Stem Cell Reports 8:787–801.

SUPPLEMENTARY MOVIE S1. Bright-field time-lapse images of mouse ESCs on E-cadherin-coated plates in maintenance medium. ESCs, embryonic stem cells.

SUPPLEMENTARY MOVIE S2. Nanog-GFP time-lapse images of mouse ESCs on E-cadherin-coated plates in maintenance medium. GFP, green fluorescent protein.

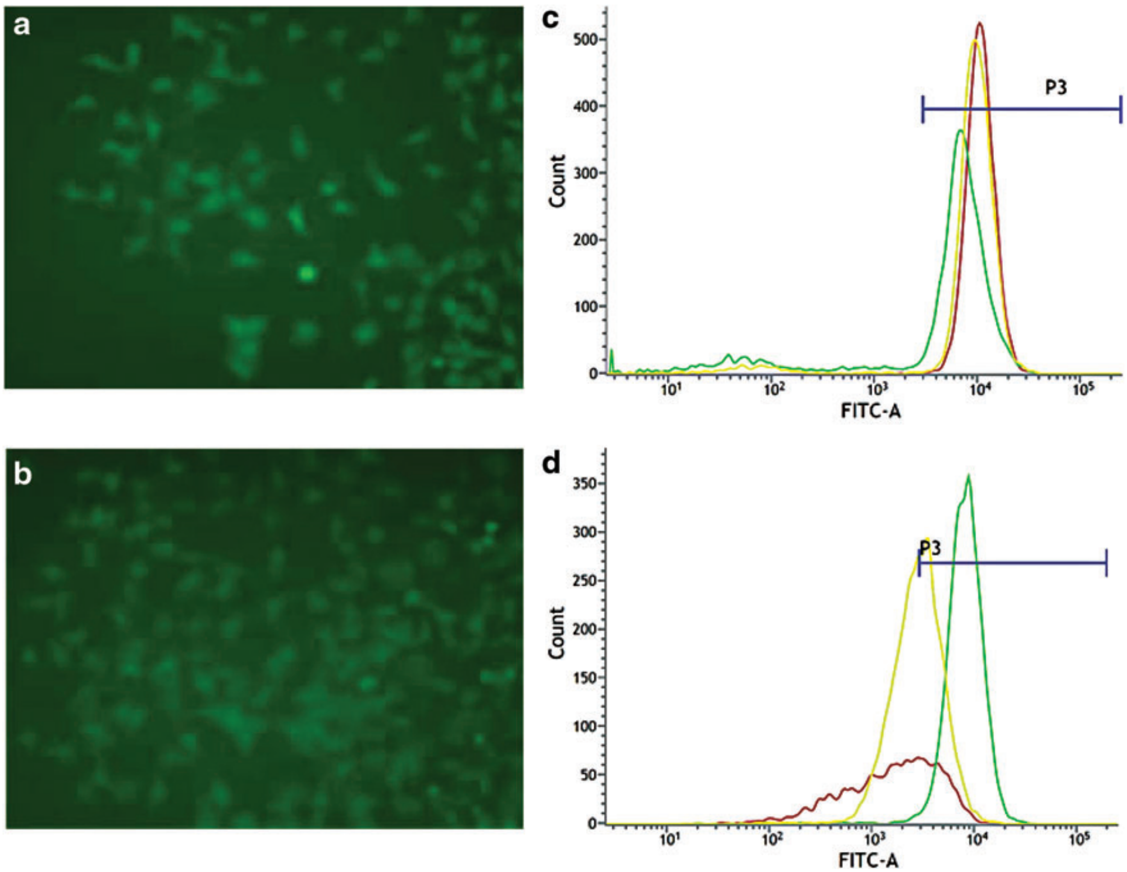

SUPPLEMENTARY FIG. S1. Nanog-GFP image and Flow Cytometry analysis of an iPSC line. **(a, b)** Fluorescent image of Nanog-GFP in maintenance medium **(a)** or differentiation medium (24 h after differentiation) **(b)**. **(c, d)**: Flow cytometry profile of Nanog-GFP cells in maintenance medium **(c)** or differentiation medium **(d)**. The profile on day 0 in maintenance medium is shown in *green*, whereas days 2 and 4 in the differentiation medium are shown as *yellow* and *red*, respectively. The cells and the culture condition are described in the Supplementary Materials and Methods section. GFP, green fluorescent protein; iPSC, induced pluripotent stem cell.

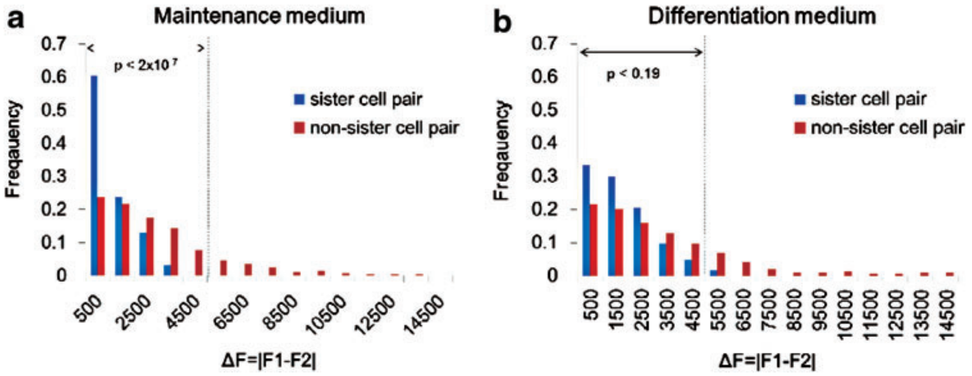

SUPPLEMENTARY FIG. S2. Sister cell analysis of Nanog-GFP intensity of an iPSC line. **(a, b)** Sixty-three sister cell pairs were measured in maintenance medium **(a)** and differentiation **(b)** medium, respectively. Distribution of the differences in fluorescence intensities between two cells in maintenance medium **(a)** and differentiation medium **(b)**. *P* values were calculated based on a chi-square test using the range of data, denoted by the *arrow*, with the other data not being used to avoid inaccuracy. The cells and the culture condition are described in the Supplementary Materials and Methods section.
