# Supplementary figures and images for: Asymmetricity Between Sister Cells of Pluripotent Stem Cells at the Onset of Differentiation
Source: Stem Cells Dev. 2018 Mar 1;27(5):347–54. doi: 10.1089/scd.2017.0113 (PMC5833898; doi:10.1089/scd.2017.0113)

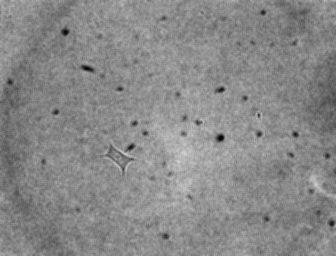

Supplement: Supplemental data [file Supp_Data.zip › Supp_Movie1.gif]

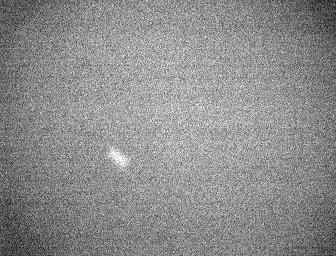

Supplement: Supplemental data [file Supp_Data.zip › Supp_Movie2.gif]
